# Identification of Urine Metabolites as Biomarkers of Early Lyme Disease

**DOI:** 10.1038/s41598-018-29713-y

**Published:** 2018-08-15

**Authors:** Adoracion Pegalajar-Jurado, Bryna L. Fitzgerald, M. Nurul Islam, John T. Belisle, Gary P. Wormser, Kathlene S. Waller, Laura V. Ashton, Kristofor J. Webb, Mark J. Delorey, Rebecca J. Clark, Claudia R. Molins

**Affiliations:** 1 Division of Vector-Borne Diseases, Centers for Disease Control and Prevention, Fort Collins, CO United States of America; 2 Department of Microbiology, Immunology and Pathology, Colorado State University, Fort Collins, CO United States of America; 3 Department of Medicine, Division of Infectious Diseases, New York Medical College, Valhalla, NY United States of America; 4 Colorado State University Health Network, Colorado State University, Fort Collins, CO United States of America; 5 Present Address: OvaCure, Copenhagen, Denmark; 6 Present Address: University of Colorado, Boulder, Colorado USA; 7 Present Address: Western Ecosystems Technology, Fort Collins, Colorado USA

## Abstract

Metabolites detectible in human biofluids are attractive biomarkers for the diagnosis of early Lyme disease (ELD), a vector-borne infectious disease. Urine represents an easily obtained clinical sample that can be applied for diagnostic purposes. However, few studies have explored urine for biomarkers of ELD. In this study, metabolomics approaches were applied to evaluate small molecule metabolites in urine from patients with ELD (n = 14), infectious mononucleosis (n = 14) and healthy controls (n = 14). Metabolic biosignatures for ELD versus healthy controls and ELD versus infectious mononucleosis were generated using untargeted metabolomics. Pathway analyses and metabolite identification revealed the dysregulation of several metabolic processes in ELD as compared to healthy controls or mononucleosis, including metabolism of tryptophan. Linear discriminant analyses demonstrated that individual metabolic biosignatures can correctly discriminate ELD from the other patient groups with accuracies of 71 to 100%. These data provide proof-of-concept for use of urine metabolites as biomarkers for diagnostic classification of ELD.

## Introduction

Lyme disease (LD) is the most commonly reported tick-borne illness in the United States, with 300,000 cases estimated to occur annually^1,2^. LD is primarily caused by the bacterium *Borrelia burgdorferi* sensu stricto and is most frequently reported from the upper Midwestern and Northeastern regions of the United States. The most common objective abnormality in patients with LD is the characteristic skin lesion called erythema migrans (EM)^3^. Approximately 70–80% of reported cases of LD have EM, often in association with non-specific symptoms such as malaise, fever, headache, fatigue, myalgia and arthralgia. LD affects both adults and children, with the highest outpatient incidence of LD reported among boys 5 to 9 years of age and in both males and females 60 to 64 years of age^2^.

All United States Food and Drug Administration (FDA) cleared laboratory diagnostic tests for LD are based on the host-antibody response to bacterial antigens, and most are applied in the standard two-tiered algorithm recommended by the Centers for Disease Control and Prevention (CDC)^4^. Several limitations exist with two-tiered testing and include a low sensitivity (29 to 40%) for early LD (ELD), the inability to distinguish past from present infection and cross-reactivity and subjectivity in the interpretation of the second-tier Western immunoblots (WB), particularly the IgM WB^5,6^. Based on the performance of serologic tests for ELD, it is recommended that patients with an EM and appropriate epidemiological risk for LD be treated without attempting laboratory confirmation of infection^7^. However, insect-bite hypersensitivity reactions and conditions that result in an EM-like rash, including Southern Tick-Associated Rash Illness (STARI) and certain cutaneous fungal infections, can be confused with an EM skin lesion^3,8^. Additionally, infections such as infectious mononucleosis (MONO) can be clinically confused with LD. Further complicating the differential diagnosis is the occurrence of false-positive serologic testing for LD in patients with MONO^9–12^. A study by Hinckley *et al*. estimated that in the United States 3.4 million diagnostic tests for LD are performed annually nationwide on approximately 2.4 million specimens^1^. This finding demonstrates that patients and physicians continue to seek laboratory confirmation of infection.

Previously, we utilized a non-targeted metabolomics approach to demonstrate that serum metabolites can accurately differentiate ELD patients from healthy controls (HC) and from patients with other diseases^13,14^. In the current proof-of-concept study, urine from patients diagnosed with ELD, MONO and HC were evaluated using a similar non-targeted metabolomics approach. Comparative metabolomics analyses between ELD and HC, as well as ELD and MONO resulted in metabolic biosignatures that accurately classified the patient groups used in this study. These results provided evidence for the development of urine-based metabolic biosignatures for ELD. Additionally, *in silico* analyses established putative structural identities for the molecular features (MFs) comprising the biosignatures and predicted metabolic pathways altered during ELD. Targeted analyses were performed to verify the identity of several MFs of altered pathways.

## Results

### Metabolic biosignatures for discriminating ELD from HC

An initial set of MFs that differed between ELD and HC or ELD and MONO was generated using the data files from LC-MS run one (Supplementary Fig. 1a). A total of 11,907 MFs for positive-ion mode and 1,755 MFs for negative-ion mode were identified by Mass Profiler Pro (MPP; Agilent Technologies, Palo Alto, CA). Those MFs with a ≥1.5-fold change between ELD and HC that also met robustness and consistency criteria (Supplementary Fig. 1a) resulted in an ELD versus HC metabolic biosignature of 1,262 MFs detected in the positive-ion mode (Data File 1). Of the 1,262 MFs, 589 had a decreased relative abundance and 673 had an increased relative abundance in ELD as compared to HC. When the same criteria were applied to MFs detected in the negative-ion mode, an ELD versus HC biosignature of 228 MFs was developed, where 94 MFs had a decreased relative abundance and 134 MFs had an increased relative abundance in ELD as compared to HC (Data File 1).

### Metabolic biosignature development to discriminate ELD from MONO

The same criteria as that applied to ELD versus HC was applied to ELD versus MONO (Supplementary Fig. 1a). This resulted in biosignatures of 1,601 MFs and 320 MFs detected in the positive-ion and negative ion-modes, respectively (Data File 1). Of the 1,601 positive ion-mode MFs, 711 MFs had a decreased relative abundance and 890 MFs had an increased relative abundance in ELD as compared to MONO. In the negative ion-mode biosignature, 151 MFs had a decreased relative abundance and 169 MFs had an increased relative abundance in ELD as compared to MONO. A total of 158 MFs overlapped between the ELD versus HC and ELD versus MONO positive-ion biosignatures, and 37 MFs overlapped between the ELD versus HC and ELD versus MONO negative-ion biosignatures.

### Metabolite identification

Presumptive chemical identification was applied to all four metabolic biosignatures created using a ≥1.5-fold abundance change (Supplementary Fig. 1b and Data File 1). Putative identifications were assigned based on the smallest ppm difference between experimental mass of the MF and the calculated monoisotopic mass of the assigned compound. Of the 1,262 MF positive-ion mode ELD versus HC biosignature, 246 MFs were assigned a putative identification by interrogation of the Human Metabolome Database (HMDB). For the ELD versus HC negative-ion mode metabolic biosignature (228 MFs), 76 MFs were assigned a putative chemical structure. Of the 1,601 MF positive-ion mode ELD versus MONO biosignature, 282 MFs had putative chemical structures assigned through HMDB and of the 320 MF negative-ion mode ELD versus MONO biosignature, 73 MFs had chemical structures assigned. It must be noted that multiple MFs were assigned the same putative identification since metabolites with similar monoisotopic mass cannot be differentiated without comparative analyses using an authentic standard.

### Creatinine assay validates LC-MS results

**Figure 1:**
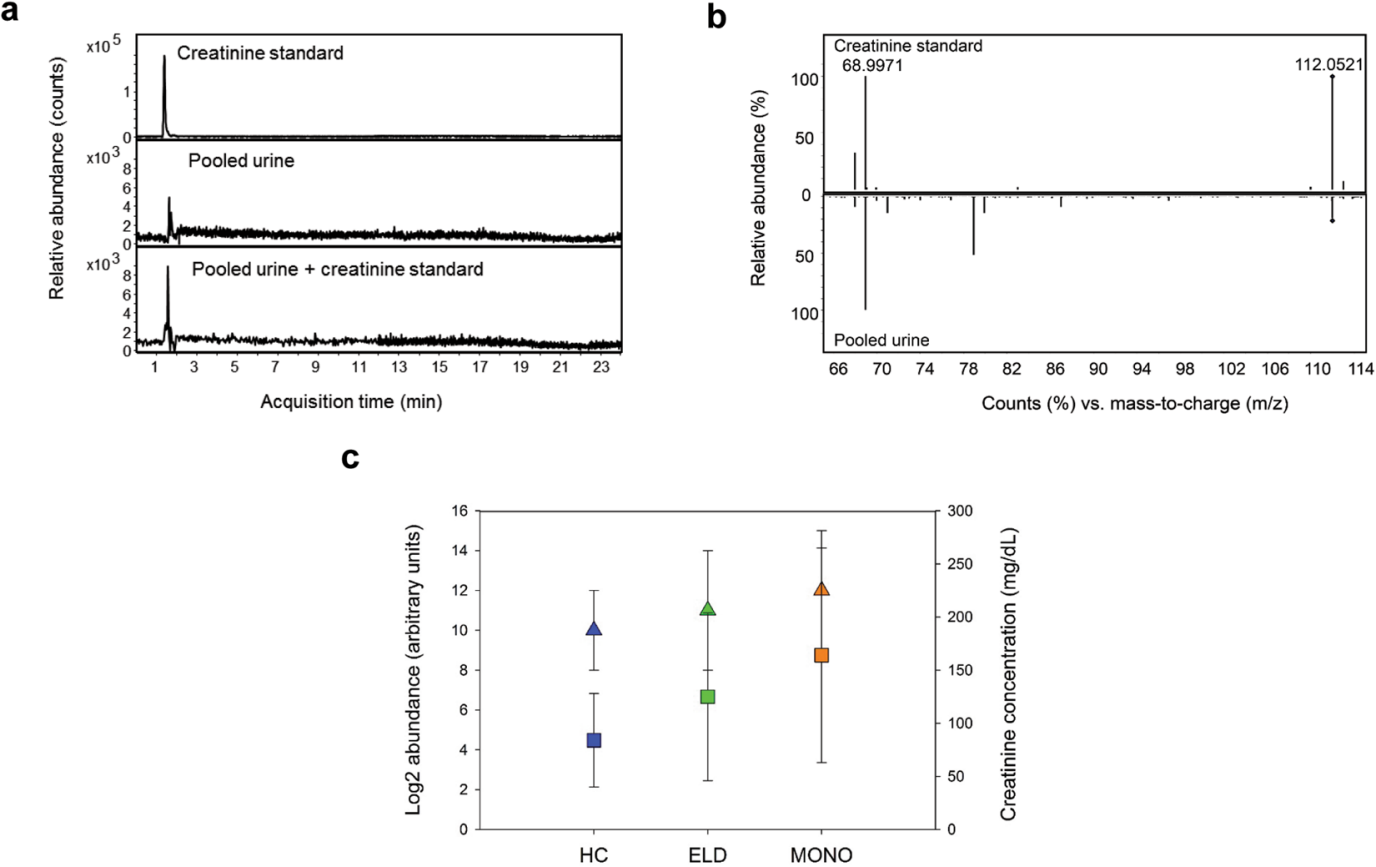
Level 1 identification and abundance confirmation of creatinine. Structural identification of creatinine (**a**,**b**) and creatinine levels (**c**). Structural identification was achieved by RT alignment (**a**) of authentic standard (top panel), the targeted metabolite in pooled urine (middle panel) and authentic standard spiked in pooled patient urine (bottom panel); and by comparison of MS/MS spectra (**b**) of the authentic standard (top panel) and the targeted metabolite in patient urine (bottom panel). Log_2_ LC-MS median abundances in HC, ELD and MONO are shown for creatinine as triangles (**c**). Creatinine concentration measured by the alkaline picrate assay are also shown in (**c**) as squares. HC are shown in blue, ELD in green and MONO in orange. For both measurement types, the mean and standard deviation from the mean are shown.

One putative metabolite identified in the negative-ion mode biosignature was creatinine. To evaluate the accuracy of our methods, this metabolite was targeted for structural identification. Creatinine was identified according to the metabolomics standards initiative definition using an authentic chemical standard^15,16^. Specifically, the MF with a monoisotopic mass of 113.0582 and RT of 1.90 min and designated as creatinine, yielded a retention time (RT) and MS/MS spectrum that was identical to the creatinine standard (Fig. 1a,b). Creatinine concentrations were also determined in all urine samples by the alkaline picrate based colorimetric assay. The average creatinine concentrations were estimated at 125 ± 79 mg/dL for ELD samples, 164 ± 101 mg/dL for MONO samples and 84 ± 44 mg/dL for HC donors (Fig. 1c). The abundance trend (calculated using the OH4 statistic) observed for this metabolite among the three groups using creatinine concentration data was HC < ELD < MONO (p = 0.004) on average. The same trend in the creatinine abundance differences was observed when relative abundances were extracted from the LC-MS data (Supplementary Fig. 1c).

### Pathways presumptively affected by ELD

**Figure 2:**
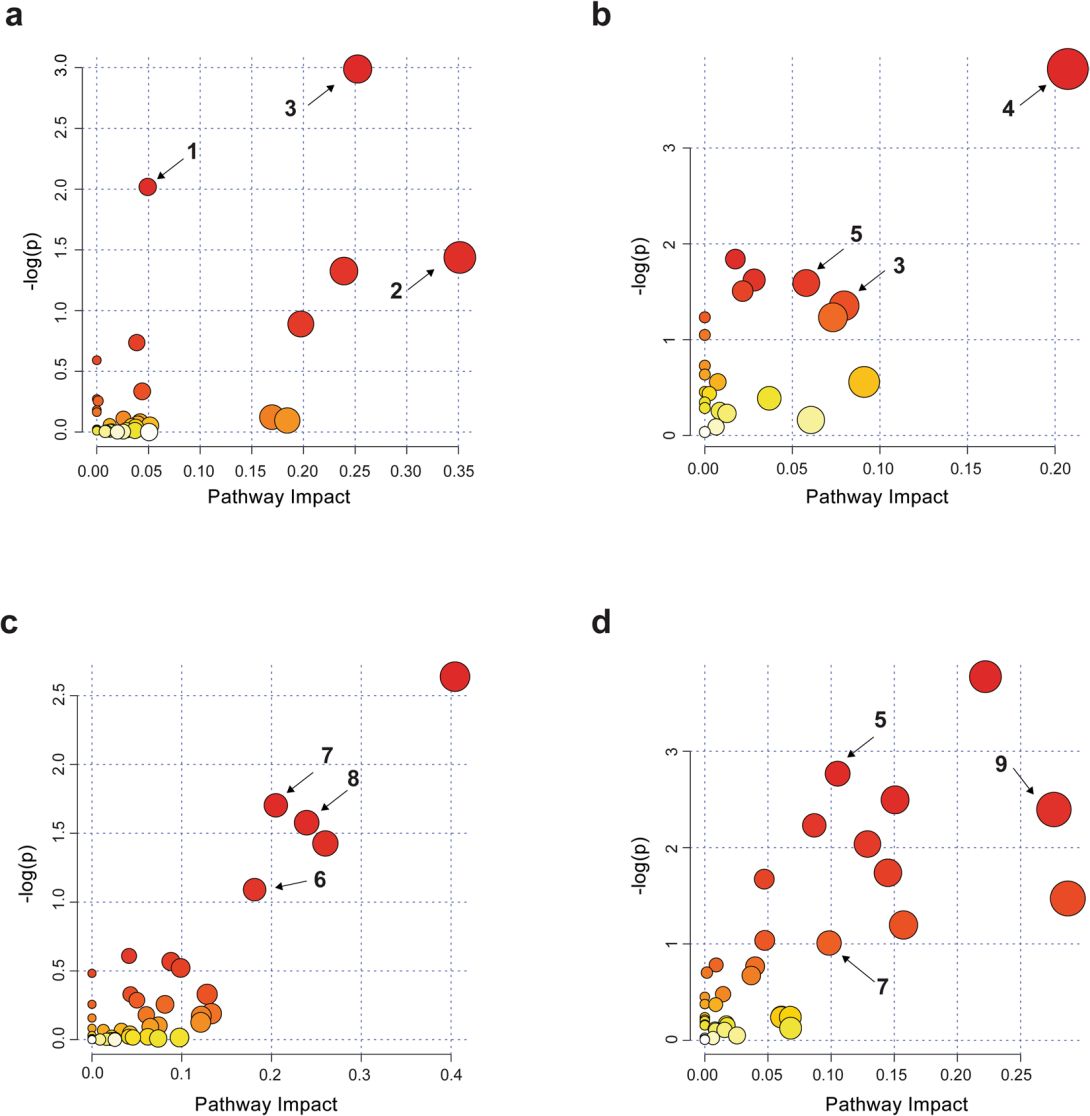
MetaboAnalyst results. MetaboAnalyst was used to identify pathways that were perturbed between (**a**) ELD versus HC using the 1,262 MF positive-ion mode metabolic biosignature, (**b**) ELD versus HC using the 228 MF negative-ion mode metabolic biosignature, (**c**) ELD versus MONO using the 1,601 MF included in positive-ion mode metabolic biosignature and (**d**) ELD versus MONO using the 320 MF negative-ion mode metabolic biosignature. The color and size of each circle is based on p-values and pathway impact values. The three top perturbed pathways based on p-values, pathway impact factor and largest number of unique masses associated with a pathway are numbered and correspond to 1. tryptophan metabolism, 2. glycerophospholipid metabolism, 3. vitamin B6 metabolism, 4. citrate cycle (TCA cycle), 5. tyrosine metabolism, 6. folate biosynthesis, 7. phenylalanine, tyrosine and tryptophan biosynthesis, 8. riboflavin metabolism and 9. biotin metabolism.

MetaboAnalyst 3.0 software (www.metaboanalyst.ca/) was applied to identify metabolic pathways potentially perturbed during ELD as compared to HC (Supplementary Fig. 1b). The most dominant pathways associated with the biosignature for ELD versus HC were tryptophan metabolism, glycerophospholipid metabolism, vitamin B6 metabolism, riboflavin metabolism, phenylalanine, tyrosine and tryptophan biosynthesis, citrate cycle (TCA cycle), tyrosine metabolism and biotin metabolism (Fig. 2a,b). Of these pathways, only vitamin B6 metabolism was identified using both the positive- and negative-ion mode data. A complete MetaboAnalyst metabolic pathway data-set for the ELD versus HC biosignatures is provided in Supplementary Table 1.

MetaboAnalyst was also used to identify metabolic pathways potentially perturbed in ELD as compared to MONO (Fig. 2c,d). The top pathway hits were folate biosynthesis, phenylalanine, tyrosine and tryptophan biosynthesis, riboflavin metabolism, one carbon pool by folate, phenylalanine, tyrosine metabolism, biotin metabolism, amino sugar and nucleotide sugar metabolism, fructose and mannose metabolism, galactose metabolism, starch and sucrose metabolism, glycolysis or gluconeogenesis and valine, leucine and isoleucine biosynthesis. Of these pathways, phenylalanine, tyrosine and tryptophan biosynthesis were identified using both the positive- and negative-ion mode data from ELD versus MONO comparisons. A complete MetaboAnalyst metabolic pathway data set for the ELD versus MONO biosignatures is provided in Supplementary Table S1.

### Directed analyses of tryptophan metabolites

**Figure 3:**
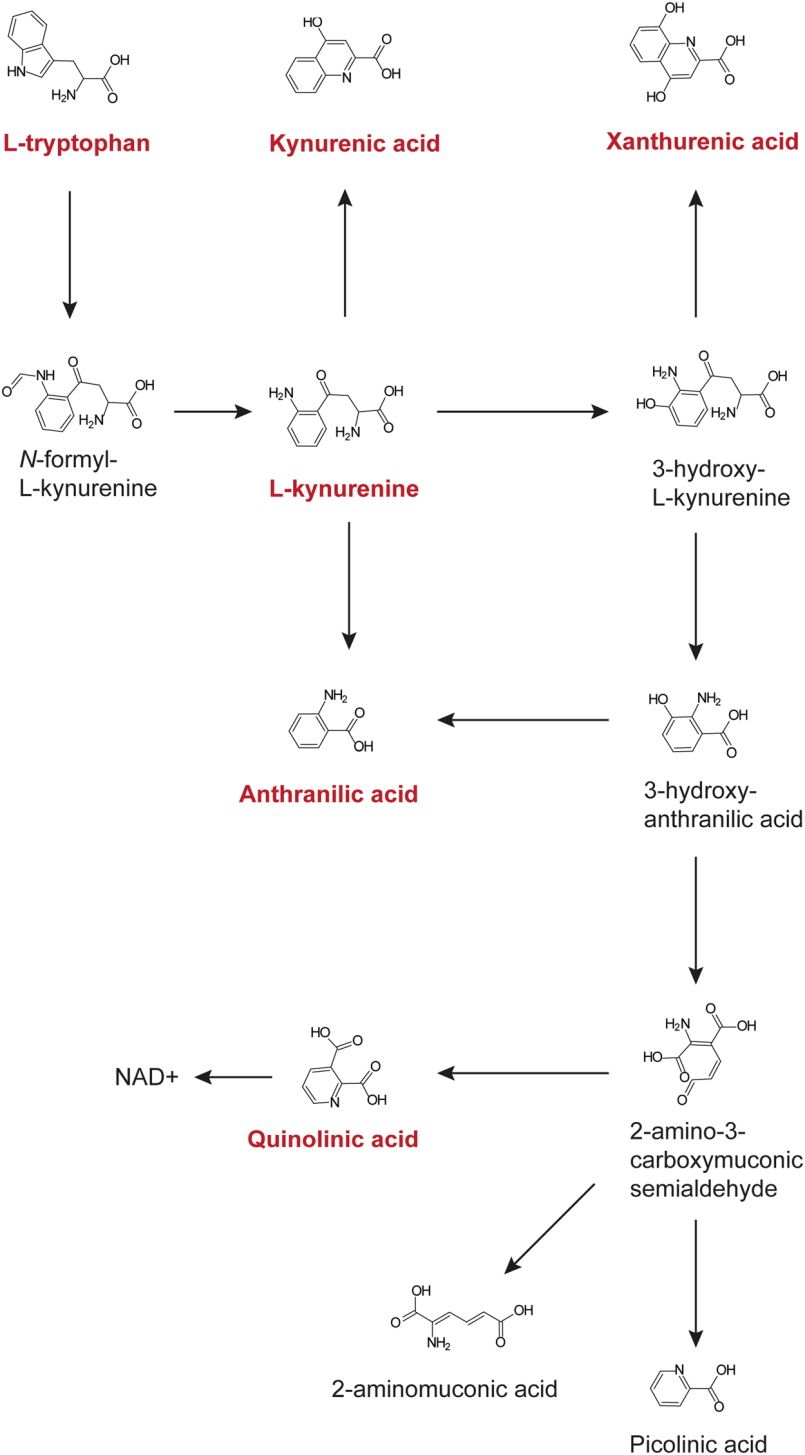
Kynurenine pathway. Tryptophan catabolism via indoleamine 2,3-dioxygenase (IDO) is shown. The metabolites depicted in red are those that were evaluated in the directed analyses.

**Figure 4:**
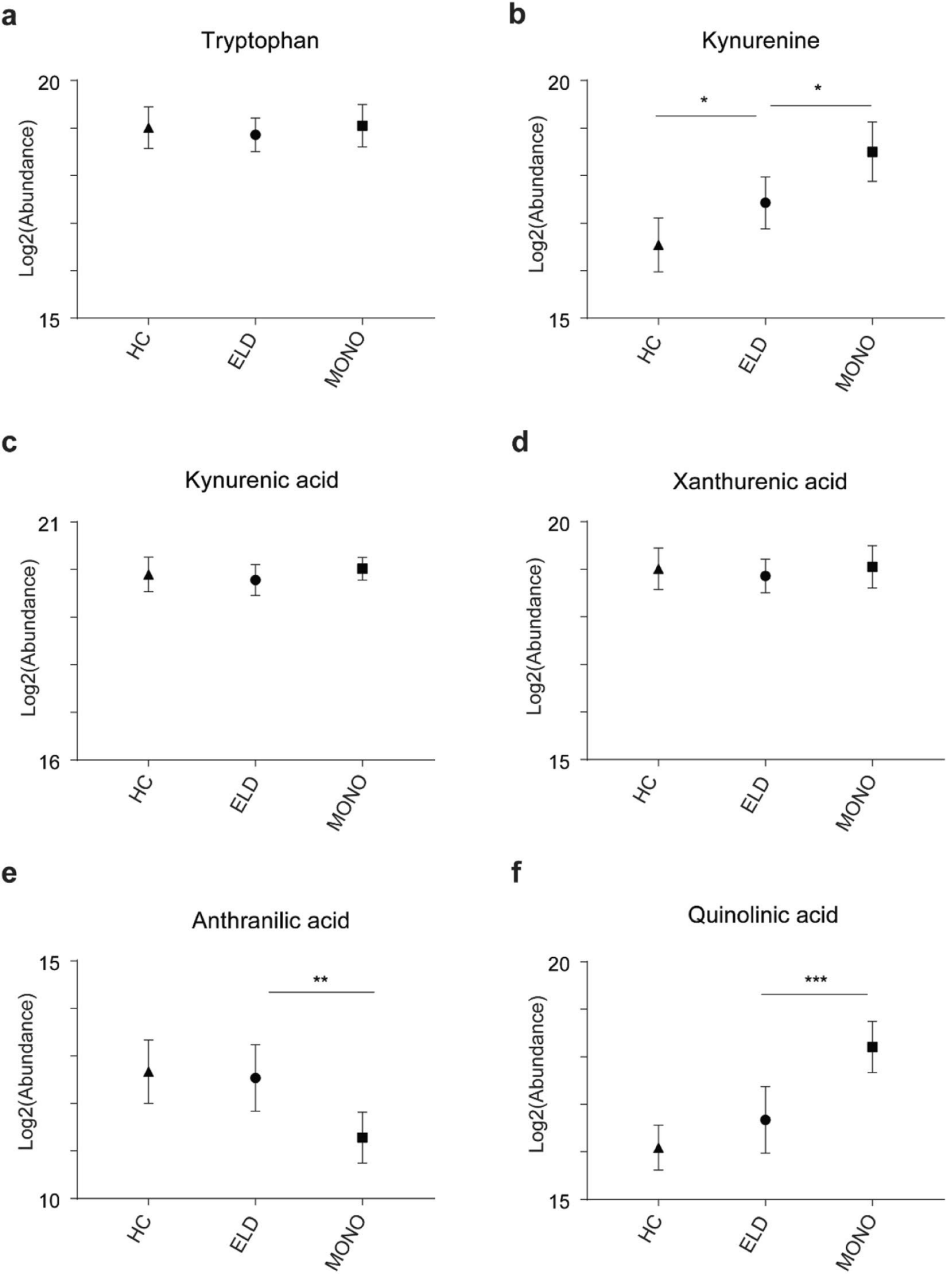
Kynurenine pathway metabolite abundances. Abundances of tryptophan (**a**) kynurenine (**b**), kynurenic acid (**c**), xanthurenic acid (**d**), anthranilic acid (**e**) and quinolinic acid (**f**) in HC, ELD and MONO patients are shown (*p < 0.05, **p < 0.01, ***p < 0.001).

Tryptophan metabolism was found to differ in both the comparisons of ELD versus HC and ELD versus MONO (Supplementary Fig. 1c and Supplementary Table S1). Since increased catabolism of tryptophan via IDO has been described in infectious diseases, including Lyme disease^17–22^, metabolites in this pathway were targeted for identification and quantitation. Xanthurenic acid, tryptophan, kynurenine, kynurenic acid, anthranilic acid, and quinolinic acid were confirmed based on their *m/z* and RT in pooled patient urine (Fig. 3 and Supplementary Figs 2–4). The normalized, log_2_ transformed abundances for kynurenine were, on average, significantly increased in ELD patients as compared to HC (p = 0.0480) and in MONO patients versus those with ELD (p = 0.0147) (Fig. 4b). Interestingly, two downstream metabolites that represent a split in the pathway (anthranilic acid and quinolinic acid) were significantly altered, on average, between ELD and MONO patients (p = 0.0092 and p = 0.0005, respectively) (Fig. 4e and f). However, the direction in the fold change for the two metabolites was opposite. Levels of tryptophan metabolites were also evaluated in ELD patients with localized versus disseminated infection. Tryptophan was significantly decreased, on average, in ELD patients with disseminated infection as compared to localized infection (p = 0.0285); however, no significant changes were observed for kynurenine, kynurenic acid, xanthurenic acid, anthranilic acid and quinolinic acid in the subgroups of ELD (Supplementary Fig. 5).

### Biosignature testing for differentiating ELD patients from HC and ELD patients from MONO patients

**Figure 5:**
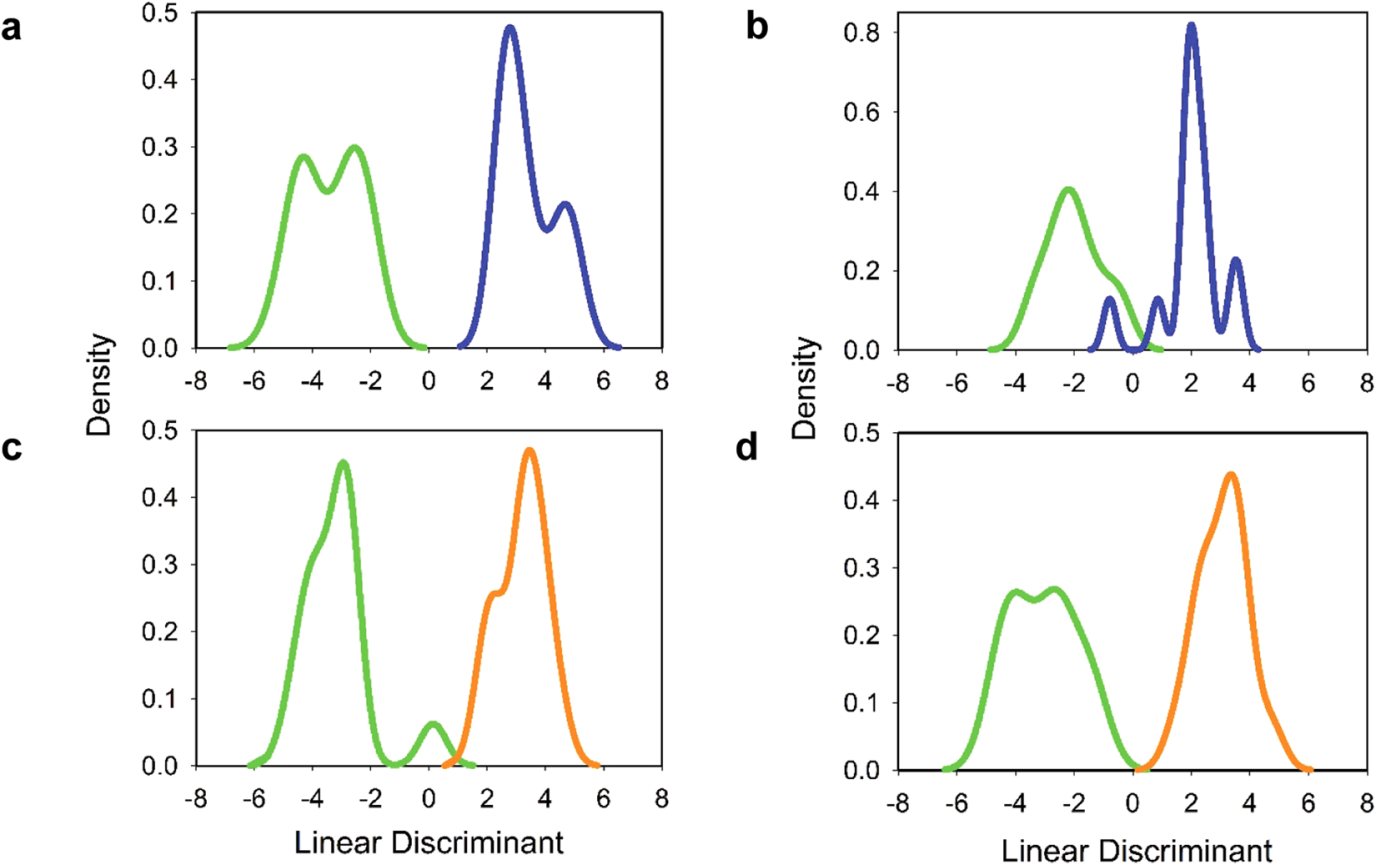
LDA analysis of ELD versus HC and ELD versus MONO. LDA analysis of (**a**) ELD (green) versus HC (blue) using the 408 MF positive-ion mode metabolic biosignature, (**b**) ELD (green) versus HC (blue) using the 113 MF negative-ion mode metabolic biosignature, (**c**) ELD (green) versus MONO (orange) using the 633 MFs positive-ion mode metabolic biosignature, and (**d**) ELD (green) versus MONO (orange) using the 164 MF negative-ion mode metabolic biosignature.

To assess whether the biosignatures developed possess diagnostic value, MFs with a ≥2.0-fold change between ELD versus HC and ELD versus MONO were evaluated by linear discriminant analysis (LDA) followed by cross-validation (Supplementary Fig. 1d). Metabolites with a ≥2.0-fold change between the two comparator groups were utilized to down-select the number of metabolites to those with a greater discriminatory value. The results of these analyses are shown in Table 1. LDA followed by leave-one-out cross-validation correctly classified 12 of 14 ELD and 12 of 14 HC when the positive-ion mode biosignature list was used (Fig. 5a). However, the negative-ion mode metabolic biosignature was slightly less accurate in classifying ELD samples (10 of 14), but was equal to the positive ion-mode biosignatures for classifying HC (12 of 14; Fig. 5b). When the metabolic biosignatures that differentiate ELD and MONO were tested 12 of 14 ELD and 14 of 14 MONO were correctly classified using the positive-ion mode metabolic biosignature and 13 of 13 ELD and 13 of 13 MONO were correctly classified when using the negative-ion mode metabolic biosignature (Table 1 and Fig. 5c,d).

**Table 1:** LDA Followed by Cross-Validation for Classification of ELD, MONO and HC groups.

LC-MS Analysis	Sample Group^^^	No. Pos. (% Pos.)			
		LDA: Leave-One-Out^#^		LDA: Leave-Two-Out*	
		ELD versus HC	ELD versus MONO	ELD versus HC	ELD versus MONO
Positive Ion Mode	HC	12 (86)	—	323 (85)	—
	ELD	12 (86)	12 (86)	324 (86)	327 (87)
	MONO	—	14 (100)	—	376 (99)
Negative Ion Mode	HC	12 (86)	—	320 (85)	—
	ELD	10 (71)	13 (93)	252 (67)	351 (93)
	MONO	—	13 (93)	—	354 (94)

Abbreviations: LDA, linear discriminant analysis; LC-MS, liquid chromatography mass spectrometry; No., number; Pos., positive; ELD, early Lyme disease; HC, healthy controls; MONO, infectious mononucleosis. ^^^A total of 14 samples were analyzed for each group. ^#^A total of 14 possible results exist for a leave-one-out analysis using 14 samples per group. *A total of 378 possible results exist for a leave-two-out analysis using 14 samples per group.

### Serologic testing

Table 2 summarizes the LD serologic testing results for the three sample groups used in this study. A total of 13 ELD patients were positive by the C6 enzyme immunoassay (EIA) and seven (50%) were positive by standard two-tiered testing using the C6 EIA followed by WB. When MONO patients were tested, two were positive by the C6 EIA and no patient was positive by standard two-tiered testing (C6 EIA followed by WB). For MONO patients only, the VIDAS whole cell sonicate (WCS) EIA was also performed, and 12 were positive or equivocal. When the VIDAS WCS EIA was used as a first-tier test in modified two-tiered testing^23^ (WCS EIA followed by C6 EIA), one patient was positive. The testing of HC sera identified two samples that were C6 EIA positive and all were negative by standard two-tiered testing. It should be noted that a negative two-tiered assay result was a requirement for inclusion as a HC.

**Table 2:** Lyme disease serologic testing results.

	No. Pos. (% Pos.)					
	C6 EIA	VIDAS WCS EIA	Marblot IgM WB	Marblot IgG WB	Two Tiered Testing	
					C6 EIA/WB	VIDAS EIA/C6 EIA
ELD (n = 14)	13 (93)	ND	7 (50)	2 (14)	7 (50)	ND
MONO (n = 14)	2 (14)	12 (86)	1 (7)	0 (0)	0 (0)	1 (7)
HC (n = 14)	2 (14)	ND	1 (7)	0 (0)	0 (0)	ND

Abbreviations: No., number; Pos., positive; WSC, whole cell sonicate; EIA, enzyme immune assay; WB, western immunoblot; ELD, early Lyme disease; MONO, infectious mononucleosis; HC, healthy controls.

## Discussion

Urine samples are readily collected in a non-invasive manner, which eliminates the risks and discomfort associated with a blood draw, an important consideration with regard to patient groups such as children and the elderly. Few LD studies have tested urine as a diagnostic specimen^24–26^ and these have had variable success and/or employed techniques that require large volumes of urine. Although we have previously provided data on the metabolic changes that can be detected in the blood of patients with ELD, no such information is available for urine. In this study, we applied a non-targeted metabolomics approach to identify metabolites in urine that differentiated between ELD and HC, as well as ELD and MONO. To maintain the focus of this work on ELD, a comparison between MONO and HC was not performed. Furthermore, due to the low number of samples, a three-way comparison between ELD, MONO and HC was not performed.

When statistical methods were applied to the abundances of the MFs comprising the ELD versus HC and ELD versus MONO biosignatures, the analyses predict that the selected MFs will be capable of differentiating between the two comparator groups with high accuracy, although it should be noted that a small sample size was used. LDA analysis showed that up to 86% (12 of 14 patients) of the ELD and HC donors were correctly classified using a leave-one out cross-validation strategy and similar results were observed when applying a leave-two out cross-validation.

Other diseases that can be confused with LD^3,27–29^ and/or that result in antibodies that are cross-reactive to *B*. *burgdorferi* antigens^9–12^ should be included as the primary control when developing and assessing novel laboratory diagnostic assays and algorithms for LD. To evaluate whether a urine-based metabolic profiling approach could differentiate ELD from a clinically relevant infection, we developed biosignatures that distinguished ELD from MONO patients. LDA analysis using the MFs of these biosignatures correctly classified 86 to 100% of the ELD and MONO samples when leave-one out cross-validation was applied; similar results were observed for leave-two out cross-validation. It is well documented that antibody-based assays for LD can result in false-positive results for MONO patients^12^. Interestingly, patients with Lyme disease can also be false-positive for primary Epstein-Barr virus infection^30,31^. The results of this study demonstrated the feasibility of using urine metabolic biomarkers as a potential diagnostic tool for ELD, even in patients with MONO.

It should be noted that there was an age difference between the ELD and MONO patient groups, and although expected given the primary age ranges for these two infections, it is a limitation of this study. Given that only 14 samples per group were evaluated, additional ELD and MONO samples as well as clinically relevant control groups are required to further validate the MFs identified in this study. Further, our study was designed to ensure the broadest coverage of metabolites, the samples were analyzed in both positive and negative ion modes. Since these proof-of-concept studies did not demonstrate one approach as better than the other, future analyses will include both. Down-selection of MFs that could be targeted for the development of a laboratory diagnostic would likely include the most robust MFs (as defined by those with the highest fold change in abundance and lowest coefficient of variation) and would have at least a level 3 identification^15^. Ideally, the number of MFs included would be 20 or less and would be analyzed using more accurate quantification, which can be achieved by applying a Multiple Reaction Monitoring (MRM) assay^32^. It is envisioned that a urine-based metabolic biosignature could be applied in a clinical laboratory using a similar premise to that which is currently used and cleared by the Food and Drug Administration (FDA) for screening inborn errors^33^.

Although urine is a preferred sample type for many diagnostic platforms, one challenge is normalization of the samples prior to LC-MS analysis due to expected physiological differences among individuals and differences in fluid intake. Urine is commonly normalized based on measured creatinine levels. This is done with the assumption that the secretion of creatinine is constant across and within individuals^34–36^. Nevertheless, when urinary creatinine varies in response to a diseased state, this normalization procedure can result in an underestimation or overestimation of the measured metabolites. This limitation has motivated several researchers to evaluate other normalization protocols and, ultimately, avoid the use of creatinine normalization in diseases that may affect creatinine secretion^34,37,38^. Based on the data from this study, creatinine levels may be affected when a patient has ELD, as well as MONO. We therefore did not use creatinine for sample normalization, instead we applied a uniform dilution approach^39^. Measuring osmolality is another normalization technique that can be applied and will be tested in future studies^40,41^.

To gain further understanding of the host response to ELD, the biosignature lists created using a MF fold change of ≥1.5 between the two comparator groups were used for metabolic pathway analyses^42^. This less stringent cutoff (≥1.5-fold change versus a ≥2.0-fold change) provided a larger number of metabolites for pathway analyses. This evaluation of metabolic pathways demonstrated that several of the putative metabolites were assigned to tyrosine metabolism; tryptophan metabolism; and phenylalanine, tyrosine and tryptophan biosynthesis. Tyrosine metabolism has been shown to be altered upon immune activation^43–46^ in several inflammatory conditions such as chronic low-grade inflammation and inflammatory bowel disease^44,47^ as well as other pathological conditions including chronic kidney failure, neurodegenerative and cardiovascular diseases, gastrointestinal disorders, heart disease, chronic fatigue syndrome and infection of lung epithelial cells^48–53^. The alteration of tyrosine and phenylalanine metabolism has also been found to occur when fatigue, fever and/or loss of appetite occur^43,51,54^. The association between tryptophan catabolism and infectious diseases including LD^17–22^ was of particular interest and led us to a directed evaluation of selected metabolites from this pathway (Fig. 3). Our results assessing metabolites of IDO mediated tryptophan catabolism corroborated the recent findings of Marques *et al*. that demonstrated EM skin lesions of LD patients had increased transcription of three genes belonging to this pathway^18^. In particular, we observed significantly increased levels of kynurenine in ELD patients in comparison to HC (Fig. 4b). However, there were no significant differences between ELD patients and HC for the other targeted metabolites (kynurenic acid, xanthurenic acid, anthranilic acid and quinolinic acid). It has been suggested that local immunosuppression and tolerance associated with increased IDO activity and production of kynurenine is important for bacterial dissemination leading to more severe forms of disease^17,18^. In line with this finding, we observed significantly decreased levels of tryptophan in ELD patients with disseminated infection versus ELD patients with localized infection (Supplemental Fig. 5). Differences in IDO mediated tryptophan catabolism between MONO and ELD were also observed in our studies. While both pathogens have been shown to induce IDO expression during infection^17–19,22^, our results indicated the possibility that the downstream branches in the pathway leading to quinolinic acid differed between ELD and MONO patients (Figs 3 and 4). This finding provided additional evidence for the utility of a urine-based metabolic biosignature to differentiate ELD and related diseases and demonstrated that urine metabolic profiling with small volumes (20 µl of urine) has the potential to provide a novel diagnostic approach for ELD.

## Methods

### Clinical samples

Previously collected paired urine (n = 42) and serum samples (n = 42) were procured from specimen repositories at New York Medical College (Valhalla, NY) and Colorado State University Hartshorn Health Center (Fort Collins, CO). All ELD (n = 14 patients) and HC (n = 14 donors) samples were from patients and donors living in New York and were collected in the same clinic using the same methods, while the MONO (n = 14 patients) samples were from patients living in Colorado, but were also collected using similar methods. Urine and sera from ELD patients were collected pretreatment at the time of clinical diagnosis (4 to 22 days following onset of illness). Six of the 14 patients with EM had multiple skin lesions. Eight of the patients with EM were positive for *B*. *burgdorferi* infection by culture (skin and blood samples tested) and/or PCR (skin and blood samples tested). ELD patients with a single EM and negative blood PCR and/or blood culture were classified as having a localized infection, and patients with multiple EMs and/or that were blood PCR and/or blood culture positive were classified as having disseminated infection. Samples from patients with MONO were collected at the time of clinical diagnosis (3 to 30 days following onset of illness), and MONO was diagnosed based on the patient’s clinical signs and symptoms, as well as a positive rapid test using the Wampole® MonoTest (Alere Inc., Waltham, MA) or a Mono-Latex test (Inverness Medical, Scarborough, ME). All MONO patients and HC reported no known history of LD. The age of patients and donors ranged from 31 to 86 years for ELD, 27 to 86 for HC and 18 to 22 for MONO (age unknown for five patients), and 71%, 50% and 64% were male, respectively.

Comorbidities were documented for ELD and MONO patients as well as healthy controls. Comorbidities included low back pain, heart disease, resected colon, hypotension, high cholesterol and cervical disk impingement among others, but a specific comorbidity did not affect more than two patients per group. Additionally, none of the patients reported known kidney or bladder disease. The samples used in this study were stored at −80 °C following collection with no additives. The Institutional Review Boards of the Centers for Disease Control and Prevention, Colorado State University and New York Medical College gave approval to conduct this study and all patients gave informed consent. All methods were carried out in accordance with the guidelines and regulations of human subjects.

### Metabolite extraction and LC-MS parameters

For LC-MS analysis, urine samples (100 µL) were diluted 1:3 (vol/vol) using LC-MS grade water (VWR, Arlington Heights, IL)^39^. Samples were centrifuged at 17,000 × g for 30 min. A total of 20 µL of diluted urine per patient was applied to an Atlantis T3, 2.1 × 150 mm, 3 µm LC column (Waters) and analyzed by an Agilent 1260 HPLC system coupled to an Agilent 6224 time-of-flight mass spectrometer (TOF) equipped with an electrospray ion source (Agilent Technologies). Metabolites were eluted with a 1 to 99% nonlinear gradient of acetonitrile in 0.1% formic acid at a flow rate of 250 µL/min. The initial 1% of acetonitrile increased to 15% after 7 min, reached 50% after 13 min and 99% within 15 min. The percent of acetonitrile returned to the initial level of 1% after 16.5 min and held at initial conditions until 22 min. Identical LC gradients were used for positive and negative ion mode analysis. The MS was operated at 310 °C gas temperature; 10 L/min drying gas nebulizer at 45 lb/in^2^; 4,000 V capillary voltage for positive-ion mode and 2800 V for negative-ion mode; 120 V fragmentation energy; 50 V skimmer and 750 V octapole RF setting. The LC-MS data for the mass range of 70 to 1700 Da were acquired at a rate of 2 spectra/s. Data were collected in both centroid and profile modes in 2 GHz extended dynamic range. LC-MS analyses were performed in duplicate over two independent LC-MS runs. Quality control urine samples consisting of all urine samples pooled were analyzed in duplicate at the beginning of each run and between every 20 samples thereafter to monitor instrument performance.

For LC-MS/MS analyses, a total of 10 µL of standard, diluted pooled urine, or diluted pooled urine spiked with standard was applied to an Atlantis T3, 2.1 × 150 mm, 3 µm LC column (Waters) and analyzed by an Agilent 1260 HPLC system coupled to an Agilent 6520 quadrupole time-of-flight mass spectrometer (Q-TOF) equipped with an electrospray ion source (Agilent Technologies). Metabolites were eluted with a 1 to 99% nonlinear gradient of acetonitrile in 0.1% formic acid at a flow rate of 250 µL/min. The gradient was the same as that used for LC-MS analyses with the exception of returning to initial condition of 1% acetonitrile at 17 min and holding at initial conditions for 23 min. The LC-MS conditions were the same as those described for the TOF analyses, with the addition of a MS/MS scan rate of 1 spectra/s. Targeted ions were fragmented using fixed collision energies (10, 20 and 40 V).

### Data analyses and metabolic biosignature development

LC-MS data were processed and biosignatures were developed as previously described with minor modifications and as shown in Supplementary Fig. 1A^13,14,35^. Briefly, MPP software version B.12.01 (Agilent Technologies) was used to identify MFs with a minimum absolute abundance of 5,000 counts and MFs present in at least 25% of the samples in at least one group. Raw LC-MS data were normalized following integration with MassHunter Quantitative Analysis software (Agilent Technologies) as previously described^14^. Minor modifications to biosignature development included the exclusion of drug metabolites and the development of two biosignature lists per ionization mode created using a ≥1.5- and a ≥2.0-fold change cutoff. Thus, a total of four metabolic biosignatures were created for each comparison (ELD versus MONO and ELD versus HC).

### Prediction and verification of MFs

The workflow utilized to assign presumptive metabolite identification of the MFs comprising the four metabolic biosignatures developed with a 1.5-fold change cutoff (two for ELD versus MONO and two for ELD versus HC) is described in Supplementary Fig. 1B. Experimental accurate masses were searched in the HMDB (http://www.hmdb.ca/) with a tolerance of 15 ppm to obtain putative metabolite identifications. Plant specific metabolites, alkaloids, non-endogenous metabolites and peptides were excluded from the results. Level identification was performed using the guidelines outlined in the Metabolomics Standards Initiative^15,16^. A RT window of ±10 sec was applied as a cut-off to match the urine metabolite with the authentic standard.

### Determination of pathways perturbed in ELD

The workflow utilized to assess the metabolic pathways altered during ELD is described in Supplementary Fig. 1B. These analyses utilized the MFs comprising the metabolic biosignatures (ELD versus HC and ELD versus MONO positive- and negative-ion modes) created using a 1.5- fold change cutoff. Pathway analysis was performed using the web-based MetaboAnalyst 3.0 software^55,56^ and MFs with presumptive HMDB identities^57^. The parameters used were the Homo sapiens pathway library, the analysis algorithms hypergeometric test and relative-betweeness centrality to estimate the relevance of a compound within a given metabolic network. The top perturbed pathways were chosen based on a pathway impact factor ≥0.1, a p value ≤ 0.5 and the largest number of unique masses associated with a pathway.

### Targeted analysis of tryptophan metabolites

A summary of the methods used for targeted analysis of tryptophan metabolites is shown in Supplementary Fig. 1C. Authentic chemical standards for tryptophan, anthranilic acid, kynurenic acid, kynurenine, quinolinic acid and xanthurenic acid were obtained from Sigma (Sigma Aldrich, St. Louis, MO). Spiked and unspiked aliquots of diluted pooled urine were analyzed by LC-MS as described. Experimental *m/z* and RT obtained from spiking experiments were used to create methods for quantitation in the MassHunter Quantitative analysis software (Agilent Technologies). Abundances were normalized according to methods used for biosignature development. Normalized log_2_ transformed abundances were plotted using GraphPad Prism 6. A D’Agostino-Pearson omnibus normality test was used to determine if residuals came from a Gaussian distribution. Statistically significant changes between ELD and MONO and ELD and HC were determined using one-way ANOVA with Sidak’s multiple comparisons test. Statistically significant changes between ELL and EDL were determined using an unpaired t-test or a Mann-Whitney test depending on normality.

### Creatinine concentration

For level 1 identification of creatinine, a creatinine authentic chemical standard was obtained from Sigma (Sigma Aldrich, St. Louis, MO). Additionally, the level of creatinine within each urine sample was measured by an alkaline picrate based colorimetric assay (Oxford Biomedical Research, Oxford, MI) according to the manufacturer’s instructions.

### Serologic testing

Serum samples obtained from all patients and donors were tested using the following first-tier EIAs: the C6 *B*. *burgdorferi* ELISA assay (Immunetics, Boston, MS) and the VIDAS WCS Lyme IgM and IgG polyvalent assay (bioMérieux, Inc., Durham, NC). Marblot IgM and IgG western immunoblots (MarDx Diagnostics, Inc., Carlsbad, CA) were used as a second-tier test. All assays were performed following the manufacturer’s instructions, and two-tiered test results were interpreted according to the recommended CDC guidelines, with the exception that all samples (regardless of the first-tier test result) were tested by both IgM and IgG Western immunoblots^4^.

### Statistical analyses

To assess the trend of creatinine concentration in ELD, HC and MONO urine samples, the OH4 statistic from Neuhäuser and Hothorn^58^ was used to test the three competing ordered alternative hypotheses of µ_HC_ = µ_EL_ < µ_MONO_; µ_HC_ < µ_EL_ = µ_MONO_; and µ_HC_ < µ_EL_ < µ_MONO_ against the null of µ_HC_ = µ_EL_ = µ_MONO_, where µ_HC_, µ_EL,_ and µ_MONO_ are the mean ranks of creatinine concentration for HC, ELD, and MONO urine samples, respectively.

For classification modeling, the metabolic biosignatures developed with a ≥2.0-fold change cutoff were used. LDA was performed on the log_2_ transformed data sets (Run 1 and 2). Within-subject variance was assumed to be constant across subjects and classification. Variance components of the common covariance matrix were estimated by mean squared errors. Leave-one-out and leave-two-out cross-validation were performed in order to estimate prediction performance. All analyses were performed using R statistical software (version 3.2.2)^59^. Supplementary Fig. 1D describes the workflow applied to assess the utility of the metabolic biosignatures for differentiating ELD from HC and ELD from MONO.

### Disclaimer

The findings and conclusions in this report are those of the authors and do not necessarily represent the official position of the Centers for Disease Control and Prevention.

## Electronic supplementary material

Supplementary Material

Dataset 1
