# Supplementary material for: Identification of Urine Metabolites as Biomarkers of Early Lyme Disease
Source: Sci Rep. 2018 Aug 15;8:12204. doi: 10.1038/s41598-018-29713-y (PMC6093930; doi:10.1038/s41598-018-29713-y)
Supplement: Supplementary file 1 — Supplementary Material [file 41598_2018_29713_MOESM1_ESM.docx]

Supplementary Materials for

**Identification of Urine Metabolites as Biomarkers of Early Lyme Disease**

Adoracion Pegalajar-Jurado, Bryna L. Fitzgerald, M. Nurul Islam, John T. Belisle, Gary P. Wormser, Kathlene S. Waller, Laura V. Ashton, Kristofor J. Webb, Mark J. Delorey, Rebecca J. Clark and Claudia R. Molins*

*Corresponding author e-mail: ard5@cdc.gov

The pdf file includes:

Fig. S1. Study design for the identification and application of differentiating molecular features (MFs).

Fig. S2. Level 1 identification of xanthurenic acid and tryptophan.

Fig. S3. Level 1 identification of kynurenine and kynurenic acid.

Fig S4. Level 1 identification of anthranilic acid and quinolinic acid.

Fig. S5. Tryptophan metabolite abundances in ELL and EDL patients.

Table S1. MetaboAnalyst results.


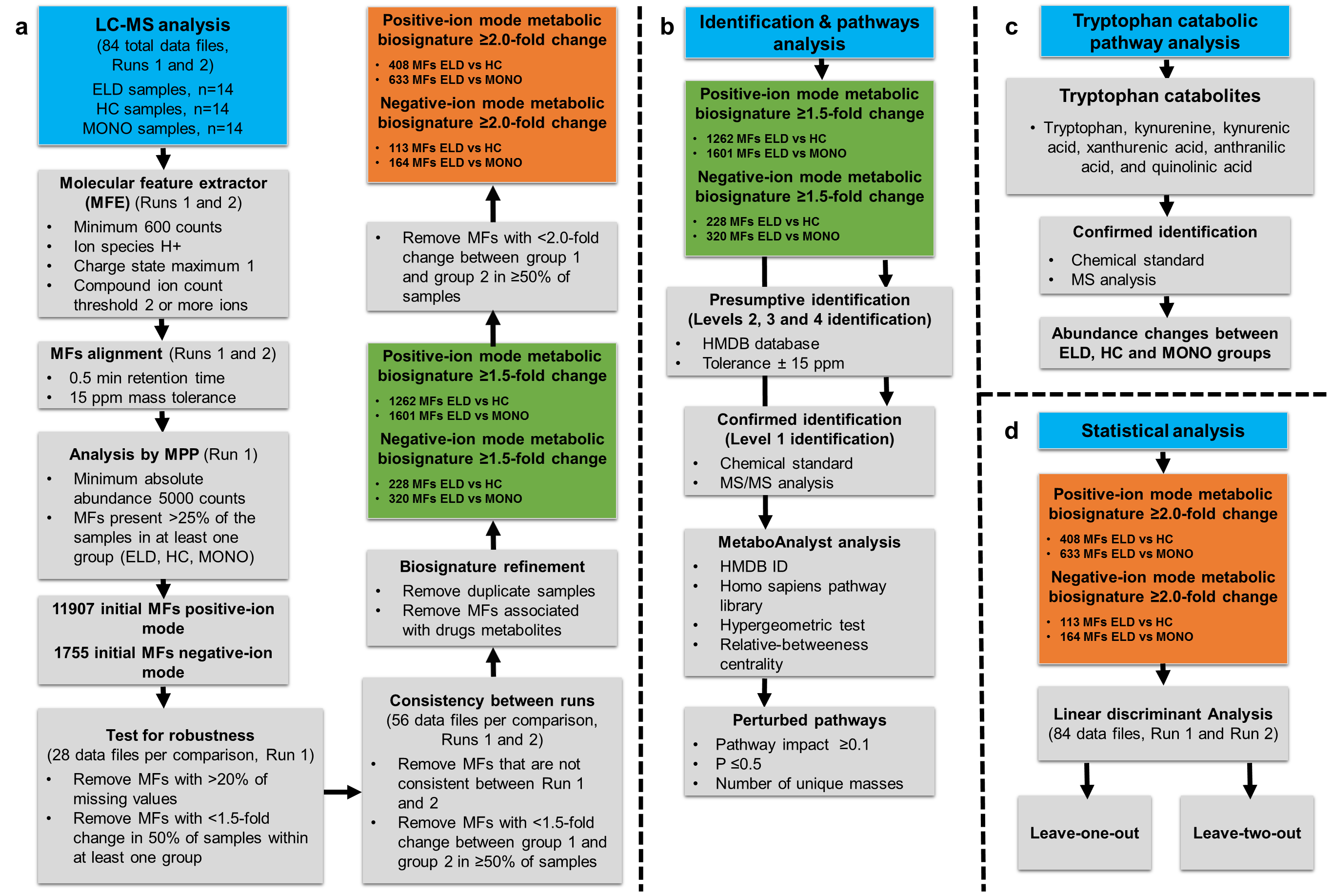


**Supplementary Figure 1. Study design for the identification and application of differentiating molecular features (MFs).**  (**a**) LC-MS data from (ELD), healthy controls (HC) and infectious mononucleosis (MONO) were used to identify lists of MFs that differed between ELD and HC, and ELD and MONO in both the positive- and negative-ion modes. The MFs were then screened for robustness and consistency and this resulted in the 1.5-fold change biosignature lists that ranged from 228 to 1,601 molecular features (MFs). A second set of biosignature lists with a 2-fold change difference was also generated for each comparison and each ionization mode. (**b**) Identification and pathway analyses were performed using the 1.5-fold change biosignature lists with HMDB and MetaboAnalyst. Level 1 confirmation was achieved using MS/MS and a chemical standard. (**c**) The tryptophan catabolic pathway was targeted and metabolites were confirmed using chemical standards. Abundances for these metabolites were compared among the three groups. (**d**) LDA and cross-validation analyses were performed using the 2.0-fold change biosignature lists. The analyses performed are shown in blue. The biosignature lists developed using a ≥1.5-fold change and ≥2.0-fold change and used in the analyses are shown in green and orange, respectively.


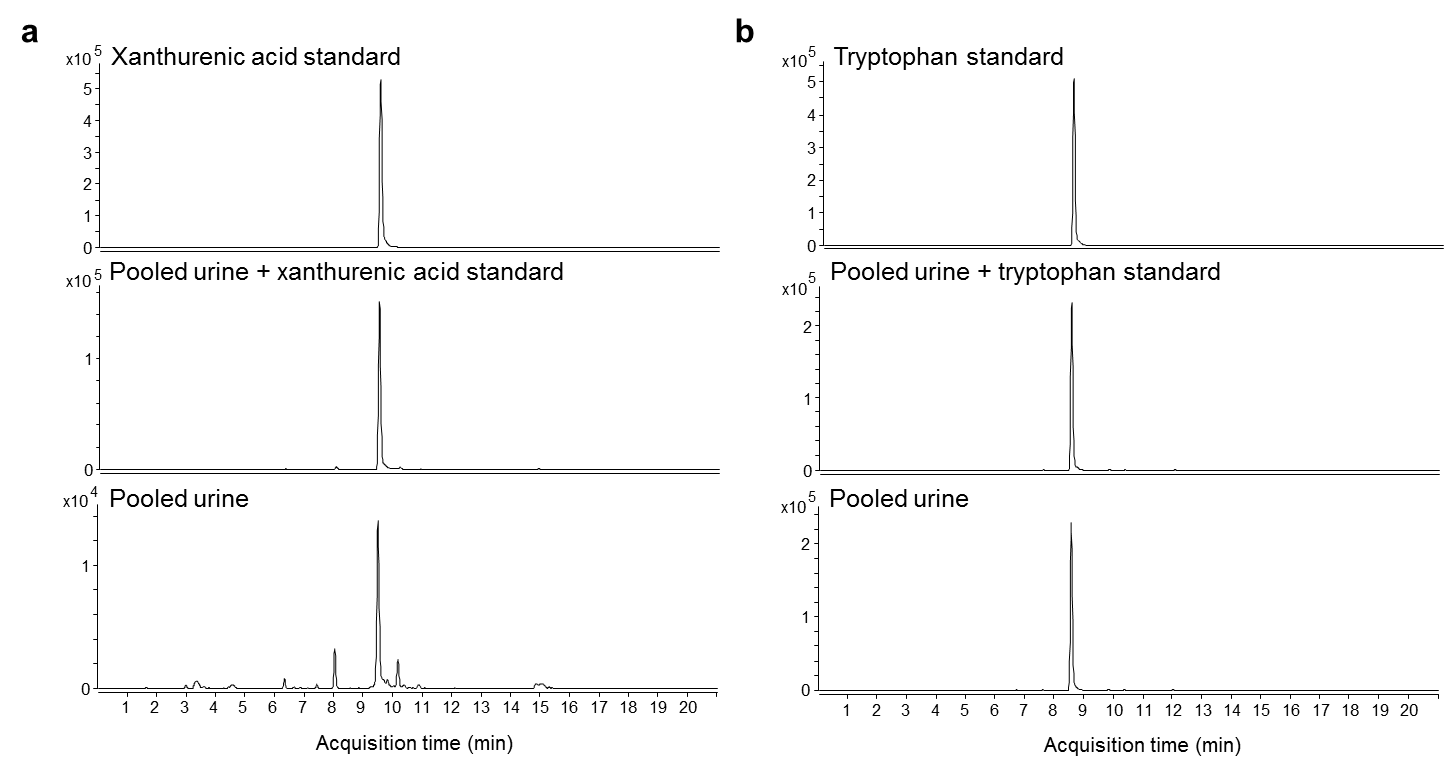


**Supplementary Figure 2. Level 1 identification of xanthurenic acid and tryptophan.** Structural identification of xanthurenic acid (**a**) and tryptophan (**b**) was achieved by RT alignment of authentic standard (upper panel) authentic standard spiked in pooled patient urine (middle panel), and the targeted metabolite in pooled urine (lower panel).


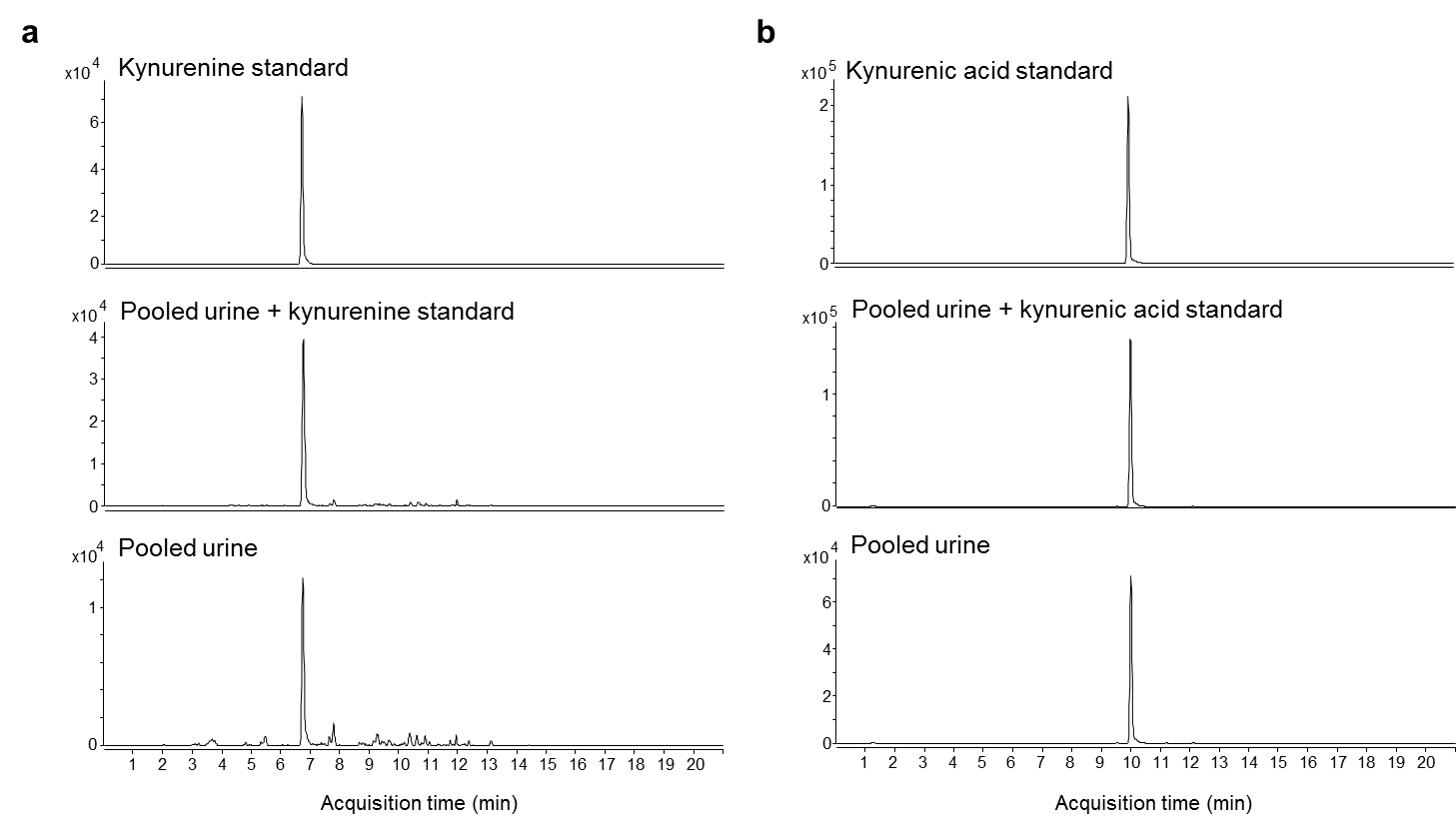


**Supplementary Figure 3. Level 1 identification of kynurenine and kynurenic acid.** Structural identification of kynurenine (**a**) and kynurenic acid (**b**) was achieved by RT alignment of authentic standard (upper panel) authentic standard spiked in pooled patient urine (middle panel), and the targeted metabolite in pooled urine (lower panel).


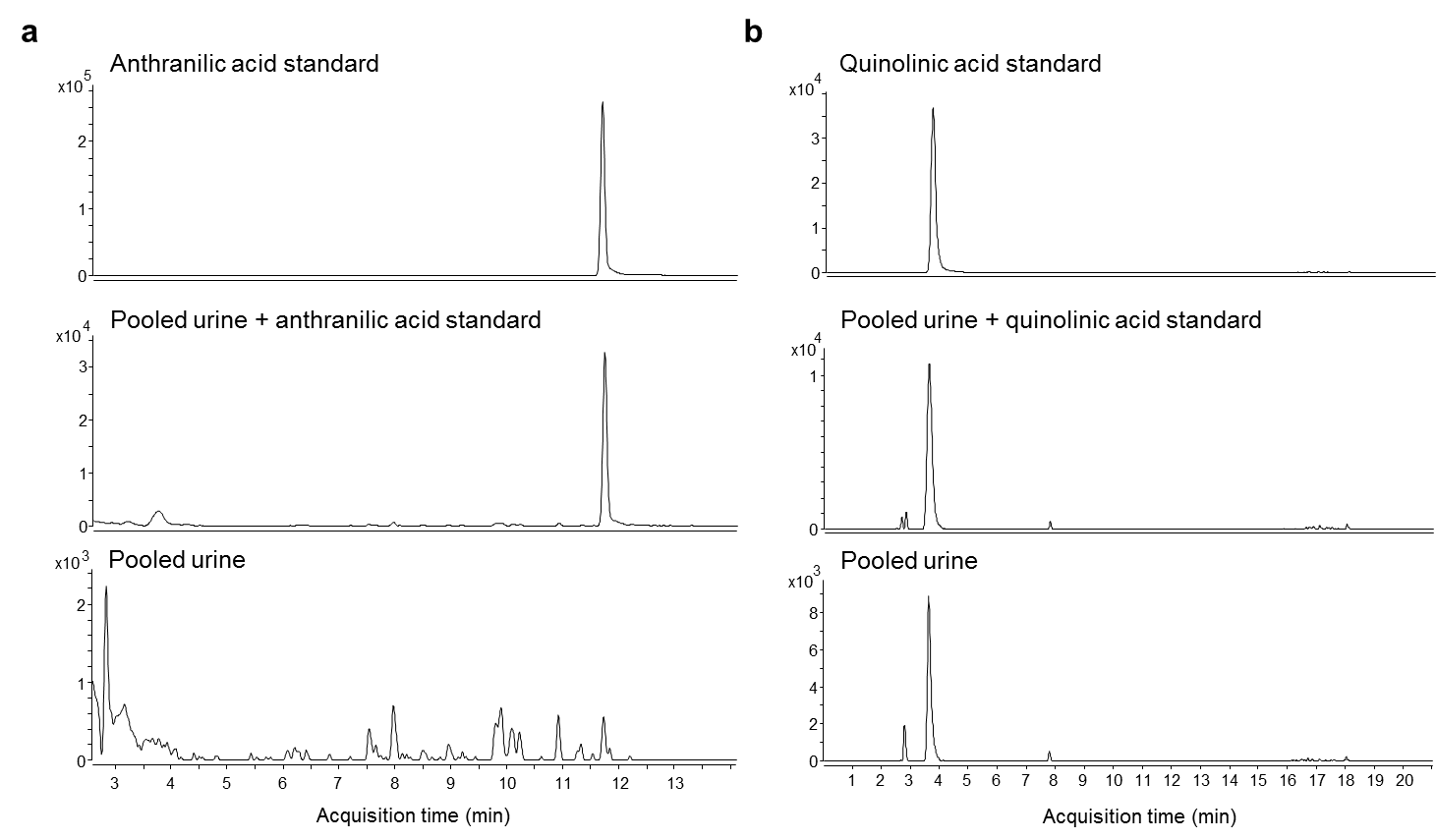


**Supplementary Figure 4. Level 1 identification of anthranilic acid and quinolinic acid.** Structural identification of anthranilic acid (**a**) and quinolinic acid (**b**) was achieved by RT alignment of authentic standard (upper panel) authentic standard spiked in pooled patient urine (middle panel), and the targeted metabolite in pooled urine (lower panel).


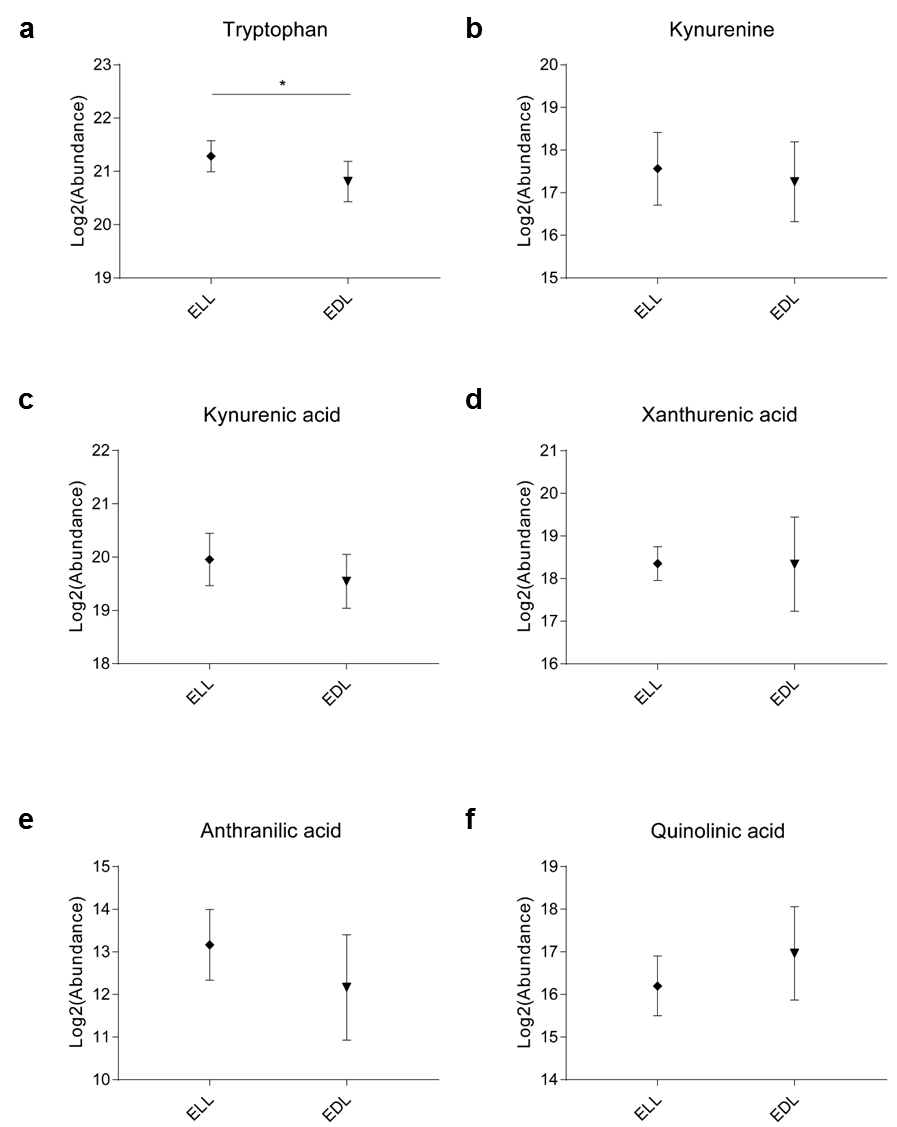


**Supplementary Figure 5. Tryptophan metabolite abundances in ELL and EDL patients.** Log_2_ LC-MS mean abundances and 95% confidence intervals in early localized Lyme disease (ELL) and early disseminated Lyme disease (EDL) patients are shown for tryptophan (**a**), kynurenine (**b**), kynurenic acid (**c**), xanthurenic acid (**d**), anthranilic acid (**e**), and quinolinic acid (**f**). A significant difference (p < 0.05) is denoted by an asterisk.

| **Table S1. Complete list of pathways identified by MetaboAnalyst for all biosignatures.** The 1262 MFs included in the ELD vs HC positive-ion mode, 228 MFs included in the ELD vs HC negative-ion mode, 1601 MFs included in the ELD vs MONO positive-ion mode and 320 MFs included in the ELD vs MONO negative-ion mode biosignature lists were analyzed using MetaboAnalyst to identify pathways perturbed in ELD patients as compared to HC and MONO. | | | | | | | | | |
| --- | --- | --- | --- | --- | --- | --- | --- | --- | --- |
| **Pathway** | **Total** | **Expected** | **Hits in MetaboAnalyst** | **Hits with unique mass** | **Raw p** | **-log(p)** | **Pathway Impact factor** | **Comparison** | **Ionization-mode** |
| Tryptophan metabolism | 79 | 7 | 10 | 8 | 0.1 | 2.02 | 0.1 | ELD vs HC | Positive-ion |
| Glycerophospholipid metabolism | 39 | 3 | 5 | 5 | 0.2 | 1.44 | 0.4 | ELD vs HC | Positive-ion |
| Vitamin B6 metabolism | 32 | 3 | 6 | 4 | 0.1 | 2.99 | 0.3 | ELD vs HC | Positive-ion |
| Riboflavin metabolism | 21 | 2 | 3 | 3 | 0.3 | 1.33 | 0.2 | ELD vs HC | Positive-ion |
| Phenylalanine, tyrosine and tryptophan biosynthesis | 27 | 2 | 3 | 2 | 0.4 | 0.89 | 0.2 | ELD vs HC | Positive-ion |
| Tyrosine metabolism | 76 | 7 | 7 | 6 | 0.5 | 0.74 | 0.0 | ELD vs HC | Positive-ion |
| One carbon pool by folate | 9 | 1 | 1 | 1 | 0.6 | 0.59 | 0.0 | ELD vs HC | Positive-ion |
| Glycosylphosphatidylinositol(GPI)-anchor biosynthesis | 14 | 1 | 1 | 1 | 0.7 | 0.34 | 0.0 | ELD vs HC | Positive-ion |
| Cyanoamino acid metabolism | 16 | 1 | 1 | 1 | 0.8 | 0.27 | 0.0 | ELD vs HC | Positive-ion |
| Lysine biosynthesis | 32 | 3 | 2 | 2 | 0.8 | 0.26 | 0.0 | ELD vs HC | Positive-ion |
| Taurine and hypotaurine metabolism | 20 | 2 | 1 | 1 | 0.8 | 0.18 | 0.0 | ELD vs HC | Positive-ion |
| Caffeine metabolism | 21 | 2 | 1 | 1 | 0.9 | 0.16 | 0.0 | ELD vs HC | Positive-ion |
| Thiamine metabolism | 24 | 2 | 1 | 1 | 0.9 | 0.12 | 0.2 | ELD vs HC | Positive-ion |
| Sphingolipid metabolism | 25 | 2 | 1 | 1 | 0.9 | 0.11 | 0.0 | ELD vs HC | Positive-ion |
| Phenylalanine metabolism | 45 | 4 | 2 | 2 | 0.9 | 0.1 | 0.2 | ELD vs HC | Positive-ion |
| Lysine degradation | 47 | 4 | 2 | 2 | 0.9 | 0.08 | 0.0 | ELD vs HC | Positive-ion |
| Steroid hormone biosynthesis | 99 | 8 | 5 | 1 | 0.9 | 0.07 | 0.0 | ELD vs HC | Positive-ion |
| Glycerolipid metabolism | 32 | 3 | 1 | 1 | 0.9 | 0.06 | 0.0 | ELD vs HC | Positive-ion |
| Terpenoid backbone biosynthesis | 33 | 3 | 1 | 1 | 1.0 | 0.05 | 0.1 | ELD vs HC | Positive-ion |
| Methane metabolism | 34 | 3 | 1 | 1 | 1.0 | 0.05 | 0.0 | ELD vs HC | Positive-ion |
| Ubiquinone and other terpenoid-quinone biosynthesis | 36 | 3 | 1 | 1 | 1.0 | 0.04 | 0.0 | ELD vs HC | Positive-ion |
| Folate biosynthesis | 42 | 4 | 1 | 1 | 1.0 | 0.02 | 0.0 | ELD vs HC | Positive-ion |
| Histidine metabolism | 44 | 4 | 1 | 1 | 1.0 | 0.02 | 0.0 | ELD vs HC | Positive-ion |
| Fructose and mannose metabolism | 48 | 4 | 1 | 1 | 1.0 | 0.01 | 0.0 | ELD vs HC | Positive-ion |
| Amino sugar and nucleotide sugar metabolism | 88 | 8 | 3 | 2 | 1.0 | 0.02 | 0.0 | ELD vs HC | Positive-ion |
| Glycine, serine and threonine metabolism | 48 | 4 | 1 | 1 | 1.0 | 0.01 | 0.0 | ELD vs HC | Positive-ion |
| Starch and sucrose metabolism | 50 | 4 | 1 | 1 | 1.0 | 0.01 | 0.0 | ELD vs HC | Positive-ion |
| Pentose and glucuronate interconversions | 53 | 5 | 1 | 1 | 1.0 | 0.01 | 0.0 | ELD vs HC | Positive-ion |
| Glyoxylate and dicarboxylate metabolism | 50 | 4 | 1 | 1 | 1.0 | 0.01 | 0.0 | ELD vs HC | Positive-ion |
| Purine metabolism | 92 | 8 | 1 | 1 | 1.0 | 0 | 0.1 | ELD vs HC | Positive-ion |
| Arachidonic acid metabolism | 62 | 5 | 1 | Mass not found | 1.0 | 0 | 0.0 | ELD vs HC | Positive-ion |
| Arginine and proline metabolism | 77 | 7 | 1 | 1 | 1.0 | 0 | 0.0 | ELD vs HC | Positive-ion |
| Porphyrin and chlorophyll metabolism | 104 | 9 | 3 | 2 | 1.0 | 0 | 0.0 | ELD vs HC | Positive-ion |
| Citrate cycle (TCA cycle) | 20 | 1 | 3 | 2 | 0.0 | 3.8 | 0.2 | ELD vs HC | Negative-ion |
| Vitamin B6 metabolism | 32 | 1 | 2 | 2 | 0.3 | 1.4 | 0.1 | ELD vs HC | Negative-ion |
| Tyrosine metabolism | 76 | 2 | 4 | 1 | 0.2 | 1.6 | 0.1 | ELD vs HC | Negative-ion |
| Biotin metabolism | 11 | 0 | 1 | 1 | 0.3 | 1.2 | 0.1 | ELD vs HC | Negative-ion |
| Phenylalanine, tyrosine and tryptophan biosynthesis | 27 | 1 | 1 | 1 | 0.6 | 0.6 | 0.1 | ELD vs HC | Negative-ion |
| Pyrimidine metabolism | 60 | 2 | 1 | 1 | 0.9 | 0.2 | 0.1 | ELD vs HC | Negative-ion |
| Ubiquinone and other terpenoid-quinone biosynthesis | 36 | 1 | 1 | 1 | 0.7 | 0.4 | 0.0 | ELD vs HC | Negative-ion |
| Glyoxylate and dicarboxylate metabolism | 50 | 2 | 3 | 2 | 0.2 | 1.6 | 0.0 | ELD vs HC | Negative-ion |
| Pentose and glucuronate interconversions | 53 | 2 | 3 | 3 | 0.2 | 1.5 | 0.0 | ELD vs HC | Negative-ion |
| Ascorbate and aldarate metabolism | 45 | 1 | 3 | 3 | 0.2 | 1.8 | 0.0 | ELD vs HC | Negative-ion |
| Starch and sucrose metabolism | 50 | 2 | 1 | 1 | 0.8 | 0.2 | 0.0 | ELD vs HC | Negative-ion |
| Primary bile acid biosynthesis | 47 | 1 | 1 | 1 | 0.8 | 0.3 | 0.0 | ELD vs HC | Negative-ion |
| Valine, leucine and isoleucine biosynthesis | 27 | 1 | 1 | 1 | 0.6 | 0.6 | 0.0 | ELD vs HC | Negative-ion |
| Arginine and proline metabolism | 77 | 2 | 1 | 1 | 0.9 | 0.1 | 0.0 | ELD vs HC | Negative-ion |
| Terpenoid backbone biosynthesis | 33 | 1 | 1 | 1 | 0.6 | 0.4 | 0.0 | ELD vs HC | Negative-ion |
| D-Glutamine and D-glutamate metabolism | 11 | 0 | 1 | 1 | 0.3 | 1.2 | 0.0 | ELD vs HC | Negative-ion |
| Butanoate metabolism | 40 | 1 | 2 | 2 | 0.3 | 1.0 | 0.0 | ELD vs HC | Negative-ion |
| Caffeine metabolism | 21 | 1 | 1 | 1 | 0.5 | 0.7 | 0.0 | ELD vs HC | Negative-ion |
| Riboflavin metabolism | 21 | 1 | 1 | 1 | 0.5 | 0.7 | 0.0 | ELD vs HC | Negative-ion |
| Alanine, aspartate and glutamate metabolism | 24 | 1 | 1 | 1 | 0.5 | 0.6 | 0.0 | ELD vs HC | Negative-ion |
| Lysine biosynthesis | 32 | 1 | 1 | 1 | 0.6 | 0.5 | 0.0 | ELD vs HC | Negative-ion |
| Tryptophan metabolism | 79 | 2 | 2 | 2 | 0.7 | 0.3 | 0.0 | ELD vs HC | Negative-ion |
| Histidine metabolism | 44 | 1 | 1 | 1 | 0.8 | 0.3 | 0.0 | ELD vs HC | Negative-ion |
| Porphyrin and chlorophyll metabolism | 104 | 3 | 1 | 1 | 1.0 | 0.0 | 0.0 | ELD vs HC | Negative-ion |
| Folate biosynthesis | 42 | 5 | 6 | 6 | 0.3 | 1.1 | 0.2 | ELD vs MONO | Positive-ion |
| Phenylalanine, tyrosine and tryptophan biosynthesis | 27 | 3 | 5 | 4 | 0.2 | 1.7 | 0.2 | ELD vs MONO | Positive-ion |
| Riboflavin metabolism | 21 | 2 | 4 | 4 | 0.2 | 1.6 | 0.2 | ELD vs MONO | Positive-ion |
| One carbon pool by folate | 9 | 1 | 3 | 3 | 0.1 | 2.6 | 0.4 | ELD vs MONO | Positive-ion |
| Phenylalanine metabolism | 45 | 5 | 7 | 3 | 0.2 | 1.4 | 0.3 | ELD vs MONO | Positive-ion |
| Tryptophan metabolism | 79 | 9 | 9 | 8 | 0.5 | 0.6 | 0.0 | ELD vs MONO | Positive-ion |
| Histidine metabolism | 44 | 5 | 5 | 5 | 0.6 | 0.6 | 0.1 | ELD vs MONO | Positive-ion |
| Ubiquinone and other terpenoid-quinone biosynthesis | 36 | 4 | 4 | 4 | 0.6 | 0.5 | 0.1 | ELD vs MONO | Positive-ion |
| D-Arginine and D-ornithine metabolism | 8 | 1 | 1 | 1 | 0.6 | 0.5 | 0.0 | ELD vs MONO | Positive-ion |
| Vitamin B6 metabolism | 32 | 4 | 3 | 3 | 0.7 | 0.3 | 0.1 | ELD vs MONO | Positive-ion |
| Pentose phosphate pathway | 32 | 4 | 3 | 2 | 0.7 | 0.3 | 0.0 | ELD vs MONO | Positive-ion |
| Nicotinate and nicotinamide metabolism | 44 | 5 | 4 | 4 | 0.8 | 0.3 | 0.1 | ELD vs MONO | Positive-ion |
| Tyrosine metabolism | 76 | 9 | 7 | 4 | 0.8 | 0.3 | 0.1 | ELD vs MONO | Positive-ion |
| Thiamine metabolism | 24 | 3 | 2 | 2 | 0.8 | 0.3 | 0.0 | ELD vs MONO | Positive-ion |
| Pantothenate and CoA biosynthesis | 27 | 3 | 2 | 2 | 0.8 | 0.2 | 0.1 | ELD vs MONO | Positive-ion |
| Inositol phosphate metabolism | 39 | 4 | 3 | 1 | 0.8 | 0.2 | 0.1 | ELD vs MONO | Positive-ion |
| beta-Alanine metabolism | 28 | 3 | 2 | 2 | 0.8 | 0.2 | 0.1 | ELD vs MONO | Positive-ion |
| Cyanoamino acid metabolism | 16 | 2 | 1 | 1 | 0.9 | 0.2 | 0.0 | ELD vs MONO | Positive-ion |
| Glycolysis or Gluconeogenesis | 31 | 4 | 2 | 2 | 0.9 | 0.1 | 0.1 | ELD vs MONO | Positive-ion |
| Terpenoid backbone biosynthesis | 33 | 4 | 2 | 2 | 0.9 | 0.1 | 0.1 | ELD vs MONO | Positive-ion |
| Citrate cycle (TCA cycle) | 20 | 2 | 1 | 1 | 0.9 | 0.1 | 0.1 | ELD vs MONO | Positive-ion |
| Caffeine metabolism | 21 | 2 | 1 | 1 | 0.9 | 0.1 | 0.0 | ELD vs MONO | Positive-ion |
| Starch and sucrose metabolism | 50 | 6 | 3 | 3 | 0.9 | 0.1 | 0.0 | ELD vs MONO | Positive-ion |
| Glyoxylate and dicarboxylate metabolism | 50 | 6 | 3 | 3 | 0.9 | 0.1 | 0.0 | ELD vs MONO | Positive-ion |
| Galactose metabolism | 41 | 5 | 2 | 2 | 1.0 | 0.1 | 0.0 | ELD vs MONO | Positive-ion |
| alpha-Linolenic acid metabolism | 29 | 3 | 1 | 1 | 1.0 | 0.0 | 0.0 | ELD vs MONO | Positive-ion |
| Purine metabolism | 92 | 10 | 5 | 4 | 1.0 | 0.0 | 0.1 | ELD vs MONO | Positive-ion |
| Pyruvate metabolism | 32 | 4 | 1 | 1 | 1.0 | 0.0 | 0.1 | ELD vs MONO | Positive-ion |
| Methane metabolism | 34 | 4 | 1 |  | 1.0 | 0.0 | 0.1 | ELD vs MONO | Positive-ion |
| Fructose and mannose metabolism | 48 | 5 | 2 | 1 | 1.0 | 0.0 | 0.0 | ELD vs MONO | Positive-ion |
| Amino sugar and nucleotide sugar metabolism | 88 | 10 | 5 | 3 | 1.0 | 0.0 | 0.0 | ELD vs MONO | Positive-ion |
| Glycerophospholipid metabolism | 39 | 4 | 1 | 1 | 1.0 | 0.0 | 0.1 | ELD vs MONO | Positive-ion |
| Arginine and proline metabolism | 77 | 9 | 3 | 3 | 1.0 | 0.0 | 0.0 | ELD vs MONO | Positive-ion |
| Porphyrin and chlorophyll metabolism | 104 | 12 | 5 | 4 | 1.0 | 0.0 | 0.0 | ELD vs MONO | Positive-ion |
| Nitrogen metabolism | 39 | 4 | 1 | 1 | 1.0 | 0.0 | 0.0 | ELD vs MONO | Positive-ion |
| Lysine degradation | 47 | 5 | 1 | 1 | 1.0 | 0.0 | 0.0 | ELD vs MONO | Positive-ion |
| Pentose and glucuronate interconversions | 53 | 6 | 1 | 1 | 1.0 | 0.0 | 0.0 | ELD vs MONO | Positive-ion |
| Glycine, serine and threonine metabolism | 48 | 5 | 1 | 1 | 1.0 | 0.0 | 0.0 | ELD vs MONO | Positive-ion |
| Metabolism of xenobiotics by cytochrome P450 | 65 | 7 | 2 | 1 | 1.0 | 0.0 | 0.0 | ELD vs MONO | Positive-ion |
| Cysteine and methionine metabolism | 56 | 6 | 1 | 1 | 1.0 | 0.0 | 0.0 | ELD vs MONO | Positive-ion |
| Pyrimidine metabolism | 60 | 7 | 1 | 1 | 1.0 | 0.0 | 0.0 | ELD vs MONO | Positive-ion |
| Aminoacyl-tRNA biosynthesis | 75 | 8 | 1 | 1 | 1.0 | 0.0 | 0.0 | ELD vs MONO | Positive-ion |
| Arachidonic acid metabolism | 62 | 7 | 1 | 1 | 1.0 | 0.0 | 0.0 | ELD vs MONO | Positive-ion |
| Tyrosine metabolism | 76 | 4 | 7 | 3 | 0.1 | 2.8 | 0.1 | ELD vs MONO | Negative-ion |
| Biotin metabolism | 11 | 1 | 2 | 2 | 0.1 | 2.4 | 0.3 | ELD vs MONO | Negative-ion |
| Phenylalanine, tyrosine and tryptophan biosynthesis | 27 | 1 | 2 | 2 | 0.4 | 1.0 | 0.1 | ELD vs MONO | Negative-ion |
| Amino sugar and nucleotide sugar metabolism | 88 | 4 | 6 | 1 | 0.2 | 1.5 | 0.3 | ELD vs MONO | Negative-ion |
| Fructose and mannose metabolism | 48 | 2 | 6 | 1 | 0.0 | 3.8 | 0.2 | ELD vs MONO | Negative-ion |
| Galactose metabolism | 41 | 2 | 3 | 1 | 0.3 | 1.2 | 0.2 | ELD vs MONO | Negative-ion |
| Starch and sucrose metabolism | 50 | 2 | 5 | 1 | 0.1 | 2.5 | 0.2 | ELD vs MONO | Negative-ion |
| Glycolysis or Gluconeogenesis | 31 | 1 | 3 | 1 | 0.2 | 1.7 | 0.1 | ELD vs MONO | Negative-ion |
| Valine, leucine and isoleucine biosynthesis | 27 | 1 | 3 | 1 | 0.1 | 2.0 | 0.1 | ELD vs MONO | Negative-ion |
| Inositol phosphate metabolism | 39 | 2 | 4 | 1 | 0.1 | 2.2 | 0.1 | ELD vs MONO | Negative-ion |
| Pentose phosphate pathway | 32 | 2 | 3 | 2 | 0.2 | 1.7 | 0.0 | ELD vs MONO | Negative-ion |
| Phenylalanine metabolism | 45 | 2 | 3 | 2 | 0.4 | 1.0 | 0.0 | ELD vs MONO | Negative-ion |
| Pentose and glucuronate interconversions | 53 | 2 | 3 | 1 | 0.5 | 0.8 | 0.0 | ELD vs MONO | Negative-ion |
| Terpenoid backbone biosynthesis | 33 | 2 | 2 | 1 | 0.5 | 0.8 | 0.0 | ELD vs MONO | Negative-ion |
| Propanoate metabolism | 35 | 2 | 2 | 1 | 0.5 | 0.7 | 0.0 | ELD vs MONO | Negative-ion |
| Ubiquinone and other terpenoid-quinone biosynthesis | 36 | 2 | 2 | 2 | 0.5 | 0.7 | 0.0 | ELD vs MONO | Negative-ion |
| Citrate cycle (TCA cycle) | 20 | 1 | 1 | 1 | 0.6 | 0.5 | 0.0 | ELD vs MONO | Negative-ion |
| Caffeine metabolism | 21 | 1 | 1 | 1 | 0.6 | 0.5 | 0.0 | ELD vs MONO | Negative-ion |
| Riboflavin metabolism | 21 | 1 | 1 | 1 | 0.6 | 0.5 | 0.0 | ELD vs MONO | Negative-ion |
| Alanine, aspartate and glutamate metabolism | 24 | 1 | 1 | 1 | 0.7 | 0.4 | 0.0 | ELD vs MONO | Negative-ion |
| Thiamine metabolism | 24 | 1 | 1 | 1 | 0.7 | 0.4 | 0.0 | ELD vs MONO | Negative-ion |
| Glyoxylate and dicarboxylate metabolism | 50 | 2 | 2 | 2 | 0.7 | 0.4 | 0.0 | ELD vs MONO | Negative-ion |
| Pyrimidine metabolism | 60 | 3 | 2 | 2 | 0.8 | 0.2 | 0.1 | ELD vs MONO | Negative-ion |
| Lysine biosynthesis | 32 | 2 | 1 | 1 | 0.8 | 0.2 | 0.1 | ELD vs MONO | Negative-ion |
| Vitamin B6 metabolism | 32 | 2 | 1 | 1 | 0.8 | 0.2 | 0.1 | ELD vs MONO | Negative-ion |
| Pyruvate metabolism | 32 | 2 | 1 | 1 | 0.8 | 0.2 | 0.0 | ELD vs MONO | Negative-ion |
| Methane metabolism | 34 | 2 | 1 | 1 | 0.8 | 0.2 | 0.0 | ELD vs MONO | Negative-ion |
| Metabolism of xenobiotics by cytochrome P450 | 65 | 3 | 2 | 1 | 0.8 | 0.2 | 0.0 | ELD vs MONO | Negative-ion |
| N-Glycan biosynthesis | 38 | 2 | 1 | 1 | 0.8 | 0.2 | 0.0 | ELD vs MONO | Negative-ion |
| Nitrogen metabolism | 39 | 2 | 1 | 1 | 0.8 | 0.2 | 0.0 | ELD vs MONO | Negative-ion |
| Butanoate metabolism | 40 | 2 | 1 | 1 | 0.9 | 0.2 | 0.0 | ELD vs MONO | Negative-ion |
| Valine, leucine and isoleucine degradation | 40 | 2 | 1 | 1 | 0.9 | 0.2 | 0.0 | ELD vs MONO | Negative-ion |
| Histidine metabolism | 44 | 2 | 1 | 1 | 0.9 | 0.1 | 0.1 | ELD vs MONO | Negative-ion |
| Ascorbate and aldarate metabolism | 45 | 2 | 1 | 1 | 0.9 | 0.1 | 0.0 | ELD vs MONO | Negative-ion |
| Lysine degradation | 47 | 2 | 1 | 1 | 0.9 | 0.1 | 0.0 | ELD vs MONO | Negative-ion |
| Primary bile acid biosynthesis | 47 | 2 | 1 | 1 | 0.9 | 0.1 | 0.0 | ELD vs MONO | Negative-ion |
| Arachidonic acid metabolism | 62 | 3 | 1 | 1 | 1.0 | 0.1 | 0.0 | ELD vs MONO | Negative-ion |
| Aminoacyl-tRNA biosynthesis | 75 | 4 | 1 | 1 | 1.0 | 0.0 | 0.0 | ELD vs MONO | Negative-ion |
| Arginine and proline metabolism | 77 | 4 | 1 | 1 | 1.0 | 0.0 | 0.0 | ELD vs MONO | Negative-ion |
| Purine metabolism | 92 | 4 | 1 | 1 | 1.0 | 0.0 | 0.0 | ELD vs MONO | Negative-ion |
| Porphyrin and chlorophyll metabolism | 104 | 5 | 1 | 1 | 1.0 | 0.0 | 0.0 | ELD vs MONO | Negative-ion |
